# Patient-perceived barriers and facilitators for risk-stratified follow-up care in lung cancer: a qualitative study

**DOI:** 10.1007/s00520-025-09868-x

**Published:** 2025-09-04

**Authors:** N. R. Moss, I. Maassen, N. E. Billingy, A. Becker-Commissaris, R. Hermens, M. Broeders, I. Walraven

**Affiliations:** 1 IQ Health Scientific Department, Raboud University Medical Center, Kapittelweg 54, Nijmegen, 6525 EP Gelderland The Netherlands; 2 Department of Pulmonology, Amsterdam University Medical Center, De Boelelaan 1118, Amsterdam, 1081 HZ Noord-Holland The Netherlands

**Keywords:** Lung cancer, Follow-up care, Risk-stratification

## Abstract

**Background:**

The shortage of healthcare professionals alongside the rising number of lung cancer survivors poses a significant challenge to current healthcare facilities. Risk-stratified follow-up care, with tailored diagnostic imaging and follow-up intervals based on a patients’ risk of recurrence, may improve clinical outcomes and help address this challenge. Our study is aimed at identifying patient-perceived barriers and facilitators for implementing this approach.

**Methods:**

A qualitative study was performed including 15 semi-structured interviews and three focus groups (*n* = 16) among lung cancer patients who completed treatment and currently receive follow-up care. Inductive and axial coding of the transcripts was performed to categorize codes into barriers and facilitators at six different levels using the Grol and Wensing framework.

**Results:**

Most barriers were identified at the organizational, economic, and political levels when shortening follow-up intervals and altering the imaging modalities due to limited available personnel, restricted imaging logistics, and financial resources. At the patient level, the most important barrier is fear of recurrence when extending follow-up intervals. Facilitators at the organizational level involved providing a direct point of contact and supportive care during risk-stratified follow-up. Overall, patients are willing to adopt risk-stratified follow-up care when sufficient evidence for its effectiveness is provided.

**Conclusion:**

We found most of the barriers, facilitators, and preferences at the organizational, economic, political, and patient level. The identified barriers and facilitators in this study can serve as a base for a strategy to implement a risk-stratified follow-up care in lung cancer care if effectiveness can be proven.

**Supplementary Information:**

The online version contains supplementary material available at 10.1007/s00520-025-09868-x.

## Background

Lung cancer survival has improved significantly as a result of the introduction of novel treatment modalities such as immunotherapy [1–3]. However, the growing number of lung cancer survivors combined with an increasing shortage of healthcare professionals (HCPs) heavily burdens the current healthcare system [4, 5]. This highlights the need to optimize the current follow-up care strategies in lung cancer care.

Currently, routine follow-up care after curative-intent therapy consists of a clinical consultation and chest computed tomography (CT) scan every six months for the first two years, followed by annual CT scans until five years after the end of curative-intent therapy [6–8]. However, this “one-size-fits-all” follow-up regimen is mainly expert based, and there is no evidence for the most optimal follow-up approach [9]. Stirling et al. concluded that the majority of lung cancer recurrences were identified within the first two years following therapy, and recurrences detected during scheduled follow-up are more likely to be asymptomatic and potentially treatable with curative intent, although the most optimal follow-up interval and imaging modality remain undetermined [10, 11]. Additionally, follow-up care for cancer patients is mainly focused on the detection of disease recurrence, often overlooking the supportive care needs of cancer patients, which may enhance health-related quality of life (HRQoL) [12–15]. It has been suggested that patients should be more directly involved in their follow-up care by including patient-reported outcome measures (PROMs) [16, 17]. Incorporating PROMs in clinical care has been shown to enhance HRQoL and improve communication between lung cancer patients and their respective HCPs [18]. Moreover, the use of PROMs in clinical care has been shown to lead to early detection of disease progression [19–21].

Therefore, a possible solution is to ensure that a novel follow-up care approach incorporates these various aspects. For instance, risk-stratified follow-up care could be used to determine the optimal follow-up interval and diagnostic imaging modality. By applying risk-stratification in follow-up care, patients can be classified as high, average, and low-risk according to each patient’s specific risk profile. This may preserve HRQoL while considering the risk of recurrence and disease [13, 22–24]. Although a few examples of risk prediction models have shown improvements in overall cancer care and follow-up in lung cancer care, the reasons for their limited adoption in clinical practice are unclear. Potential factors include limited external validation and insufficient support of both HCPs and patients [12, 24–27].

Currently, there is a lack of knowledge regarding the patient’s perspective to adopt such a risk-stratified follow-up care approach. Hence, it is essential to investigate and integrate the needs and preferences of lung cancer patients in risk-stratified follow-up care since this may lead to increased patient satisfaction and potentially clinical outcomes [5, 28, 29]. Therefore, the aim of this study is to report patients’ views on current follow-up care and to identify barriers and facilitators for implementing risk-stratified follow-up care in lung cancer patients treated with curative intent.

## Material and methods

### Study design

A qualitative study was performed including both semi-structured and focus group (FGs) interviews to identify patients’ views on current follow-up care and patient-perceived barriers and facilitators for implementing a risk-stratified follow-up in lung cancer patients. The study is reported according to the consolidated criteria for reporting qualitative research (COREQ) [30]. The study protocol was approved by the research ethics committee METC Oost-Nederland (nr. 2023–16243) and conducted in accordance with the Declaration of Helsinki.

### Population and recruitment

For this study, we approached stage I–IV lung cancer patients who participated in the SYMPRO-Lung study and gave consent to be invited for future studies [31]. In short, the SYMPRO-Lung study evaluated the clinical effect of patient-reported symptom monitoring in lung cancer patients in a multicenter trial including three university hospitals and 11 non-university hospitals in The Netherlands. From this study, 322 of the 515 SYMPRO-Lung study participants gave written consent for additional research, and they were approached by e-mail or telephone to participate in a semi-structured interview or FG. Patients were eligible to participate in the current study when the following criteria were met: the patient (1) was treated for lung cancer in the past 2 years, (2) had sufficient Dutch language proficiency, and (3) was aged ≥ 18 years [31]. Patients who expressed interest in participating were selected according to their preference for the most convenient location and availability to participate in a FG. If they were unable to participate in a FG, they were asked to take part in a semi-structured interview instead.

### Procedure

The semi-structured interviews and FGs took place from July to August 2023 and were carried out in Dutch by three researchers (IM, NM, and IW). Interviews and FGs were conducted until no new themes or topics were identified, and data saturation was achieved. The interviews were conducted face-to-face or online using Microsoft Teams video conference. The FGs, each consisting of four to eight patients, were conducted on site at a local (non)university hospital or university in The Netherlands near the residences of patients. Prior to the interviews or FGs, all patients completed a questionnaire consisting of sociodemographic and health-related information (e.g., lung cancer type, stage, treatment) and provided written informed consent. During focus groups and interviews, all patients initially received a brief explanation of risk-stratified follow-up care. This included an overview of clinical criteria used to define risk categories for follow-up care, such as follow-up intervals and diagnostic imaging modality. To standardize the direction of the discussions, a combined topic guide for the interviews and focus groups was developed based on the literature, experience within the research team, and the theoretical framework of Grol and Wensing [32]. We used the Grol and Wensing theoretical framework to structure the topic guide and identify barriers and facilitators at six different levels: innovation, patient, individual professional, social context, organizational, economic, and political level. The topic guide included questions on patients’ experiences with current follow-up care, perspectives on risk-stratified follow-up care, and barriers and facilitators for implementation of risk-stratified follow-up care based on the six different levels of the Grol and Wensing framework. The topic guide was pilot tested twice prior to the start of the study and adjusted according to feedback. The complete version of the topic guide, translated to English, is available in Supplementary file 1. All semi-structured interviews and FGs were audio-recorded, transcribed verbatim, and anonymized.

### Data analysis

Two researchers (IM and NM) independently coded the transcripts using Atlas.ti Scientific Software Development GmbH version 23.1.1. First, all interviews were fully read, and phrases were descriptively labeled by open coding. Second, comparable descriptive codes were combined and redefined into specific subthemes and themes using axial coding [33]. The theoretical framework of Grol and Wensing was used to identify barriers and facilitators at six levels. To generate agreement, consensus meetings with the research group were held to establish a coherent level of interpretation, decide on category phrasings and to validate preliminary findings. IBM SPSS Statistics 29 was used to analyze the baseline patient characteristics.

## Results

### Patient characteristics

Data saturation was reached after a total of three FGs (*n* = 16 patients) and 15 semi-structured interviews. Self-reported patient characteristics are presented in Table 1. The study cohort consisted of 31 patients, with a mean age of 68 years (range 51–81 years), and 13 (42%) of the interviewed patients were female. Most patients were diagnosed with non-small lung cancer (NSCLC) (*n* = 17, 55%) and were predominantly treated in a non-university hospital (*n* = 28, 90%). Most patients (*n* = 20, 65%) were treated with chemotherapy, while only three (10%) patients received targeted therapy.

**Table 1:** Baseline patient characteristics

	Total patients (*n* = 31)
Age in years, mean (range)	68 (51–84)
Female, ***n*** (%)	13 (42)
Level of education according to Statistics Netherlands (CBS), ***n*** (%)	
Low	9 (29)
Medium	11 (36)
High	11 (36)
Lung cancer type, ***n*** (%)	
NSCLC	17 (55)
SCLC	5 (16)
Other	1 (3)
Unknown/missing	8 (26)
Lung cancer TNM stadium, ***n*** (%)	
Stadium I	6 (19)
Stadium II	7 (23)
Stadium III	10 (32)
Stadium IV	7 (23)
Unknown/missing	1 (3)
Type of treatment, ***n*** (%)	
Chemotherapy	20 (65)
Radiotherapy	18 (58)
Surgery	15 (48)
Immunotherapy	14 (45)
Targeted therapy	3 (10)
Type of hospital, ***n*** (%)	
University hospital	3 (10)
Non-university hospital	28 (9

Thematic analysis was used to report patients’ views on current follow-up care and identify barriers and facilitators for implementing risk-stratified follow-up care in lung cancer patients.

### Patient perspectives on current follow-up care

In general, patients reported follow-up consultations every three to four months in the first year. These consultations also included a chest CT scan. Overall, patients were satisfied with their current follow-up care and had confidence in the expertise of their respective HCPs. Nonetheless, patients experienced a lack of supportive care regarding physical functioning and psychosocial care during follow-up.

*"There is actually no aftercare, because nobody asks how are you are doing between [consultations]. You just sit at the table with the pulmonologist or oncologist, who discusses what he sees on the scan, and then says there are no abnormalities found and I’m very happy." (Interview).*

Additionally, patients reported inadequate continuity of care, including frequent changes in HCPs administering their follow-up care and the absence of a direct point of contact. Moreover, patients mentioned that they failed to receive sufficient details or an explanation for any modifications that may be required regarding their follow-up interval.

Overall, patients valued HCPs who exhibit good bedside manners, including the expression of compassion, clear communication, and efficient coordination when administering follow-up care. In particular, patients valued the strong decisiveness and communication skills of specialized nurses. Specialized nurses were considered more accessible, resulting in patients feeling fewer barriers to ask questions and discuss various topics.

*"[with a specialized nurse] I talk about my skin, my social life, my own life and life with my partner, and more about the medical side with a doctor." (FG 1).*

### Barriers and facilitators

An overview of the most important barriers and facilitators according to the six levels of the Grol and Wensing framework is presented in the Supplementary Table 1. Supportive quotes per level can be found in Supplementary Table 2.

#### Level 1: innovation

Patients had a positive view on risk-stratified follow-up care and recognized its potential to personalize current lung cancer follow-up care. In addition, patients were optimistic towards less frequent follow-up visits for low-risk patients. Nonetheless, patients emphasized the importance of ad hoc consultations, in person or by phone, and via e-consults, in case of alarming symptoms. Another mentioned facilitator for the acceptance of risk-stratified follow-up care was the need for a direct point of contact during follow-up care. Patients expressed a favorable outlook towards the involvement of a specialized nurse during follow-up care and suggested that their involvement may facilitate the implementation of risk-stratified follow-up care.

#### Level 2: patient

In general, patients indicated to feel strained and anxious about upcoming follow-up consultations and feared a poor outcome especially during the early years of follow-up. Because of this fear, patients expressed the need to receive follow-up care consultations, mainly because it provides clarity on their current health status.

Regarding follow-up intervals, patients differed in their preferences: some preferred to come as often as possible to enhance their feeling of safety, while others preferred to extend the follow-up intervals due to the enhanced feeling of returning back to their normal life.

*"[Extended follow-up intervals] In itself it is nice because you can resume and return back to normal life." (interview).*

Patients indicated that the main barrier for the extension of follow-up intervals would be that it potentially increases the chance of missing a progression or recurrence of lung cancer.

*"Then if after a year they detect something, makes you think why did I agree to extending the follow-up interval? [lung cancer] it’s a silent killer, you don’t feel it and that’s the disadvantage." (FG 3).*

Patients also emphasized that it is crucial to include preferences in order to assist the implementation. As a result, they expressed that gradually extending follow-up intervals would be beneficial in the early stages of implementing risk-stratified follow-up care.

Patients did not express a preference for any particular kind of diagnostic imaging, although they do feel that more extensive diagnostic imaging offers a greater sense of security.

*"With a whole-body scan, they check everything. Because what if it is somewhere other than in your lungs? [With a PET-CT scan] they will see that too" (Interview).*

Nonetheless, patients stated to value and trust the expertise of their respective HCP to determine the optimal patient’s follow-up care. Consequently, they were willing to adopt risk-stratified follow-up care when sufficient evidence for its effectiveness is provided by their respective HCPs. 

#### Level 3: individual professional

According to patients, the most important barrier at the individual professional level was the expected increase in workload associated with shortening time between the follow-up consultations.

*"I think it would be more difficult if there are more consults, because of more post-treatments, more hospital visits. Therefore, it increases the burden on the healthcare providers." (interview).*

Moreover, patients mentioned that HCPs may feel less involved in providing care when extending follow-up intervals. Nonetheless, they suggested that HCPs may find that adopting risk-stratified follow-up care enables them to be well prepared for consultations, leading to improved patient management and reducing the need for subsequent interventions. Consequently, patients stated that more experience, knowledge, and evidence served as important facilitators for implementing risk-stratified follow-up care at the individual professional level. Additionally, they indicated that it remained essential to incorporate the HCPs’ clinical view when conducting risk-stratified follow-up care.

#### Level 4: social context level

Perceived barriers at the social level were mainly present when extending the follow-up intervals. Relatives may have trouble with prolonged uncertainty between each follow-up visit. In contrast, patients indicated that longer follow-up intervals, including fewer hospital visits, could also positively impact the emotional well-being of their relatives and themselves.

#### Level 5: organizational level

The main barrier was related to the organizational work structure and capacity. Patients indicated that when follow-up intervals are shortened, the current limitations in healthcare resources, such as restricted personnel availability and constrained hospital and diagnostic imaging logistics, may be pressured.

*"[Risk-stratified follow-up care] is wonderful, but it costs more time, more personnel and more money, that we don’t have." (FG 1).*

However, they mentioned that extending the follow-up intervals could alleviate the workload of HCPs and reduce pressure on hospital logistics.

The key facilitator at the organizational level was the continuity of care as perceived by patients. Moreover, integrating improved supportive care facilities regarding overall fitness and psychosocial care during follow-up was perceived as a facilitator for implementing risk-stratified follow-up care regardless of the varying follow-up intervals.

Patients stated that detailed guidance on the application of the risk-stratified follow-up care should be provided. In addition, they noted that the risk-stratified follow-up care should align with the current structure of clinical care practice, including a short waiting period between clinical results and a patient’s clinical follow-up consultations to facilitate the implementation. Furthermore, they suggested that data exchange through connection of electronic patient records is necessary for facilitating effective communication and coordination among their respective HCPs when administering risk-stratified follow-up care with various follow-up intervals and type of imaging.

#### Level 6: economic and political level

Patients expressed the feeling that privacy laws in healthcare are too strict, complicating the information exchange between HCPs in hospitals and primary care, which may be a barrier for effective communication and coordination when implementing risk-stratified follow-up care. Furthermore, they expressed the view that risk-stratified follow-up care should remain affordable and accessible for all lung cancer patients. Nonetheless, they expressed that extending follow-up intervals could reduce healthcare costs and stressed that the implementation of risk-stratified follow-up care should be proportionate in comparison to potential beneficial healthcare outcomes.

*"[implementing risk-stratified follow-up care] For the entire costs of healthcare that is also getting out of hand and if you can reduce this in a sensible way without enormously increasing the risks, you should do it." (interview).*

## Discussion

The findings of this study provide a unique insight into patient perspectives on current follow-up care and into barriers and facilitators for implementing risk-stratified follow-up in lung cancer care. The most important barrier at the patient level was the fear of missing progressive disease or recurrence when follow-up intervals are extended. Barriers identified at organizational, economic, and political levels, such as limited personnel, imaging logistics, and financial constraints, may further hinder altering the follow-up intervals and diagnostic imaging modalities. Facilitators were primarily found at the patient and organizational level. Patients emphasized the need for comprehensive information and evidence on the effectiveness of risk-stratified follow-up care, a preference for more nurse-led follow-up care, and improved supportive care facilities. In addition, they highlighted the necessity of having a dedicated direct point of contact during follow-up care for themselves and their relatives.

To our knowledge, this is the first study to identify barriers and facilitators for adopting risk-stratified follow-up care in lung cancer patients. Previous studies in breast cancer patients found similar barriers, such as the need for detailed information on risk communications and concerns on accessibility of care due to potential additional costs [34–36]. In a study by Voets et al., a personalized follow-up care approach in breast cancer patients observed that assumed low-risk patients received more surveillance compared to high-risk patients [37]. This indicates that it is difficult for HCPs to assess risk and determine the optimal follow-up approach without an objective risk prediction model. Additionally, studies have shown that a subgroup of lung cancer patients experience asymptomatic recurrences, and these patients may benefit from a more frequent and scheduled follow-up approach [10, 11, 38]. Although certain risk prediction models have been developed to improve clinical outcomes for lung cancer survivors through risk-based surveillance, they have not been widely implemented [24–26, 39]. Therefore, to facilitate implementation of a risk-stratified follow-up care approach, including altering the follow-up care intervals and diagnostic imaging modalities, we found that HCPs must give patients a thorough explanation and comprehensive information while administering clinical follow-up care to reassure them of their current state of health. However, patients’ preferences for follow-up intervals varied, and no preference for a specific diagnostic imaging was expressed. They further show flexibility in imaging modalities and intervals when risk-stratified follow-up care is administered consistently and continuously.

Moreover, we found that current follow-up care does not meet patients’ supportive care needs. Patients reported feeling anxious before and during follow-up consultations, regardless of adjusting the follow-up intervals. Previous research has shown that unmet supportive care needs are associated with decreased HRQoL, with fear of recurrence as a prominent emotional unmet need [40–43]. Nonetheless, in a study by Siebel et al., lung cancer patients stated that follow-up consultations also form a sense of reassurance, which contributes to improved social-emotional well-being [44]. Similarly, in order to improve cancer follow-up care, research including patients in head and neck cancer as well as breast cancer patients emphasized the need of incorporating psychological support alongside reassurance regarding detection of recurrence during follow-up care [45, 46]. While routine follow-up in a patient-initiated follow-up approach is seen as reassuring, it is also associated with increased anxiety, travel requirements, waiting time, and additional costs [47]. This trade-off between comfort and inconvenience is crucial, considering that we identified that including patients’ needs and preferences are important to facilitate implementation of risk-stratified follow-up care.

A limitation of this study is the patient recruitment method, where we included lung cancer patients who participated in the SYMPRO-Lung study [31]. This may introduce selection bias, as the participants represent a relatively healthy cohort, having been in follow-up care for over 3 years. Therefore, the study findings may not reflect the perspectives of all lung cancer patients worldwide. However, the SYMPRO-Lung study was a pragmatic trial including lung cancer patients with a wide range of demographic characteristics and treatment outcomes increasing the transferability of our findings. Another limitation is the inclusion of patients with all stages of lung cancer and various treatment types, which may introduce variability in their follow-up needs and preferences. This approach was chosen to capture a comprehensive range of patient experiences, which is essential for developing a risk-stratified follow-up care model. Additionally, patients'self-reported demographic and clinical data may introduce recall bias. Nonetheless, our findings on patients’ needs and preferences are valuable for understanding lung cancer patient perspectives on follow-up care. Furthermore, two different researchers conducted interviews and FGs. Despite the use of a topic guide to standardize the FGs and interviews, variation in the results may be present. To minimize variability, the two researchers pilot-tested the topic guide and practiced interviews together. A key strength of this study is the combined use of FGs and interviews, which enabled us to capture diverse patient perspectives, accommodating both patients who preferred a group setting and those who favored individual interviews, thus improving the validity of our findings. Therefore, our results provide valuable insights into the patients’ perspective on the implementation of risk-stratified follow-up care, which may serve as an effective approach to reduce unnecessary follow-up care and thereby decrease costs.

## Conclusion

In conclusion, this study identified patient-perceived barriers and facilitators for implementing risk-stratified follow-up in lung cancer care. Overall, patients were willing to adopt risk-stratified follow-up care when sufficient evidence for its effectiveness is provided. Risk-stratification may have great potential to improve the follow-up care of lung cancer patients by addressing patients’ needs and improving the allocation of healthcare resources based on individual risk profiles. Additionally, to accomplish a greater degree of support, further research should investigate the HCPs’ perspective on implementing risk-stratified follow-up in lung cancer care.

## Supplementary Information

Below is the link to the electronic supplementary material.

Supplementary file1 (DOCX 16.2 KB)

Supplementary file2 (DOCX 18.3 KB)

Supplementary file3 (DOCX 17.0 KB)

Supplementary file4 (DOCX 25.8 KB)

## Data Availability

The data is available from the corresponding author upon reasonable request.
