# Supplementary material for: Patient-perceived barriers and facilitators for risk-stratified follow-up care in lung cancer: a qualitative study
Source: Support Care Cancer. 2025 Sep 4;33(10):833. doi: 10.1007/s00520-025-09868-x (PMC12411594; doi:10.1007/s00520-025-09868-x)
Supplement: Supplementary file 1 — Supplementary file1 (DOCX 16.2 KB) [file 520_2025_9868_MOESM1_ESM.docx]

Supplementary file 1. Interview and focus group topic guide

**Brief explanation risk-stratified follow-up care**

Currently, after completion of initial treatment every lung cancer patient receive follow-up care every three to six months in the first year. This consists of a consultation with their respective healthcare provider and diagnostic imaging, such as a CT-scan, PET-CT-scan or x-ray.

In this study we investigate a more personalized approach, using risk-stratification to determine the most optimal follow-up approach.

We want to discuss whether it is possible and desirable to personalize follow-up care using clinical criteria (e.g. type of treatment, symptoms, performance status) to determine the optimal time between consultations and the type scan based on a patient's individual risk of recurrence or disease progression. For example, a high-risk patient should receive more frequent follow-up consultations and a low-risk patient should receive less frequent follow-up.

**General discussion topics**

1. How do patients perceive the current follow-up care after initial treatment for lung cancer?
2. How can risk-stratified follow-up care for lung cancer patients meet patients' needs?
3. According to involved patients, what are barriers and facilitators for implementing risk-stratified follow-up care?

**The six domains of the Grol and Wensing framework**
Innovation level

1. What is the best format to deliver risk-stratified follow-up?
2. How much (additional) time do you think the use of risk-stratified follow-up care may take up?

Patients level

1. How would you feel about your follow-up care being personalized by using the risk-stratified follow-up care?
   1. Do you see any advantages? If so, which?
   2. Do you see any disadvantages, if any?
2. Do you think there are solutions to these problems?
   1. If so, how could we achieve this?
3. What would it take for you as a patient to implement risk-stratified follow-up care? / What would you need?
4. How would you feel about a change of time intervals between appointments with the use of risk-stratified follow-up care? For example, a longer or shorter interval?
5. How would you feel if the type of imaging test changed, for example a PET-CT instead of CT-scan, with the use of risk-stratified follow-up care?

Individual professional level

1. Considering the health care providers you have seen in your treatment journey. What do you think your own health care providers may think of risk-stratified follow-up care?
2. What do you think health care providers may encounter when implementing or using risk-stratified follow-up care for lung cancer patients?
   1. What are possible disadvantages they may experience?
   2. Do you think there are solutions to these problems?
   3. If so, how could we accomplish this?
3. What could be of assistance to health care providers in implementing risk-stratified follow-up care?

Social context level

Consider: a fellow patient and/or their family and friends

1. How do you think people in your social environment feel about risk-stratified follow-up care?
   1. What problems do you expect?
   2. Do you think there are solutions to these problems?
   3. If so, how could we achieve this?
2. What could be of assistance for the implementation of risk-stratified follow-up care?

Organizational level

Consider: a healthcare institution or hospital and healthcare professionals

1. Looking at the healthcare institution or hospital where you are treated, would it be possible to introduce risk-stratified follow-up care?
2. Which issues do you think they might encounter?
   1. How are these issues to be resolved?
3. What could help the implementation of risk-stratified follow-up care?

Economic and political level

Consider: legislation, insurance coverage or reimbursement of care.

1. In implementing risk-stratified follow-up care in your healthcare institution, do you anticipate any issues regarding money?
2. In implementing risk-stratified follow-up care in your healthcare institution, do you anticipate any problems in the area of legislation?
3. What is required in terms of money and/or legislation to facilitate the introduction of risk-stratified follow-up care in your healthcare institution?
   1. What problems do you anticipate?
4. Are the identified problems surmountable?

If so, how could we achieve this?
