# Supplementary material for: Patient-perceived barriers and facilitators for risk-stratified follow-up care in lung cancer: a qualitative study
Source: Support Care Cancer. 2025 Sep 4;33(10):833. doi: 10.1007/s00520-025-09868-x (PMC12411594; doi:10.1007/s00520-025-09868-x)
Supplement: Supplementary file 2 — Supplementary file2 (DOCX 18.3 KB) [file 520_2025_9868_MOESM2_ESM.docx]

Supplementary Table 1. The barriers and facilitators table by Grol and Wensing framework

| Table 1a. Barriers | | | | | |
| --- | --- | --- | --- | --- | --- |
| Innovation | Patient | Individual professional | Social context | Organizational | Economic and political |
| Feasibility  - High risk:  more follow-up consultations due to shorting follow-up intervals | Attitude/feeling  - Fear of unstable disease or poor prognosis - Low risk:  increase of fear by extending the follow-up periods  - High risk:  more frequent follow-up consults, increase pressure on private life and stress  Needs - Lack of continuity of care  - Lack of a direct point of contact | Knowledge  Hard to classify a patient using risk-stratification  Attitude/feeling  - Low risk: less involvement in patient follow-up  - High risk: Increase of workload due to a different work manner - Fear of missing a diagnosis | Attitude/feeling - Low risk:  more uncertainty between each follow-up consultations  - High risk:  more frequent hospital visits and increase of burden | Workload  - High risk:  Increases the workload of HCP’s due to more frequent follow-up consultations.  Work capacity Limited healthcare resources including: - available staff - hospital logistics - financial restrictions | Legislation  Strict privacy laws  Economic constraints Restricted reimbursement of supportive care and travel expenses |

Supplementary Table 1a. Barriers identified using by Grol and Wensing framework. Abbreviations: Healthcare Practitioner (HCP).

| Table 1b. Facilitators | | | | | |
| --- | --- | --- | --- | --- | --- |
| Innovation | Patient | Individual professional | Social context | Organizational | Economic and political |
| Feasibility  - Ad hoc consultations in case of alarming symptoms  - A direct point of contact  - More nurse led follow-up care  Attractiveness  - Low risk: less frequent follow-up saves time | Needs - Incorporate feelings and wishes - Provide sufficient evidence of effectiveness  Feelings - Low-risk: enhanced patients’ feeling of returning to their normal lives - High risk: timely detection of disease progression, and feeling secure and in control  Preferences Slowly extend follow-up intervals | Communication - Exhibit good bedside manners using clear explanations - Specialized nurses exhibit clear communication and decisiveness  Motivation More preparedness for follow-up consults  Needs Incorporate HCP’s clinical perspective | Communication - Provide a good patient – HCP relationship  Motivation - Low risk:  enhanced emotional well-being  Needs A direct point of contact  Feelings - Trust in the HCPs expertise and knowledge  - Nurse-led follow-up is more accessible | Work structure - Provide supportive car - Provide nurse-led follow-up - Provide accessible data sharing with electronic patient records - Good communication and coordination between specialties  Knowledge and information - More experience, knowledge and evidence  - Provide sufficient information  Capacity - Provide continuity of care including more staff - Limit waiting time between clinical results and consults  Workload - Low risk:  less frequent follow-up consults and decreases the workload. | Legislation  Facilitate medical data sharing  Finance and policy Provide proportional, accessible and affordable follow-up care |

Supplementary Table 1b. Facilitators identified using by Grol and Wensing framework. Abbreviations: Healthcare Practitioner (HCP).
