# Supplementary material for: Patient-perceived barriers and facilitators for risk-stratified follow-up care in lung cancer: a qualitative study
Source: Support Care Cancer. 2025 Sep 4;33(10):833. doi: 10.1007/s00520-025-09868-x (PMC12411594; doi:10.1007/s00520-025-09868-x)
Supplement: Supplementary file 3 — Supplementary file3 (DOCX 17.0 KB) [file 520_2025_9868_MOESM3_ESM.docx]

Supplementary Table 2. Supportive quotes table per Grol and Wensing framework level

| Table 2. Supportive quotes | | | |
| --- | --- | --- | --- |
| **Level** | **Theme** | **Barriers** | **Facilitators** |
| **Innovation** | Motivation  Attitude and feelings | “The new standard protocol includes a question: how are we going to check this man or woman? There are perhaps three options. Well, they don't have those options now and they must think about that.” (interview) | “It is very important to know how to reach someone between appointments. It remains important no matter the time intervals, that you know who to call and when.” (FG 1) |
| **Patient** | Attitude and feelings | “Then if after a year they detect something, make you think why did I agree to extending the follow-up interval? [lung cancer] it’s a silent killer, you don’t feel it and that’s the disadvantage.” (FG 3) | “It's essentially reassurance: guys, it's done. And then you can deal with it for a while and of course you dread that scan, but that does not outweigh the result that you hear the outcome of that scan. That's the point, so, we all dread that scan, because I notice it, I just feel it, but everyone wants that scan, because we want to know if we are clean.” (FG 2)  “I think it’s important to have trust in the provided follow-up care, [….] whether you have to go every six weeks or every twelve weeks, I think you should be able to trust on the doctor's knowledge.” (interview) |
| **Individual professional** | Attitude and feelings  Motivation | “I think it would be more difficult if there are more consults, because of more post-treatments, more hospital visits. Therefore, it increases the burden on the healthcare providers.” (interview) | “[Risk-stratified follow-up care could be implemented] If questions can be answered correctly and are well formulated and substantiated.” (interview) |
| **Social context** | Communication  Needs | “During my illness, my wife was stressed, she couldn’t do it, she was on the sideline working very hard” (FG 1) | “Just kindly explain what is going on and don’t beat around the bush, be clear this is what we’re going to do.” (interview)  “[With a specialized nurse] I talk about my skin, my social life, my own life and life with my partner, and more about the medical side with a doctor.” (FG 1) |
| **Organizational** | Work structure and capacity | “[Risk-stratified follow-up care] is wonderful, but it cost more time, more personnel and more money, that we don’t have.” (FG 1) | “What I personally missed is, if you look at the total picture, for example I had psychological and physical care as part of aftercare, but the hospital could not offer both” (FG 3)  “If they [patients] can go to three months instead of six weeks with this number of patients then it naturally reduces the workload because you simply have fewer patients. The longer the period between, the less pressure there is on care.” (interview) |
| **Economic and political** | Legislation  Finance | “Privacy is something I think it’s defeating its purpose” (Interview) | “[Implementing risk-stratified follow-up care] For the entire costs of healthcare that is also getting out of hand and if you can reduce this in a sensible way without enormously increasing the risks, you should do it.” (interview) |

Supplementary Table 2. Supportive quotes table per Grol and Wensing framework level
